# Digital health: contributions from Nursing

**DOI:** 10.1590/1518-8345.0000.4407

**Published:** 2024-11-04

**Authors:** Ana Estela Haddad, Swedenberger Barbosa, Paulo Eduardo Guedes Sellera, Marcelo D’Agostino

**Affiliations:** ^1^ Ministério da Saúde, Secretaria de Informação e Saúde Digital, Brasília, DF, Brazil; ^2^ Ministério da Saúde, Secretaria Executiva, Brasília, DF, Brazil; ^3^ Organização Pan-Americana da Saúde/Organização Mundial da Saúde, Departamento de Evidência e Inteligência para Ação em Saúde, Brasília, DF, Brazil

**Figure:**
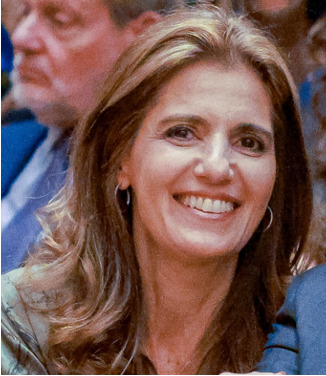

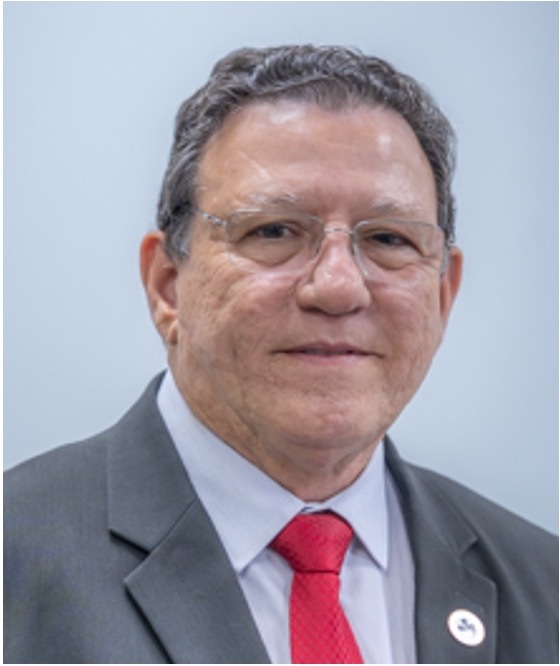

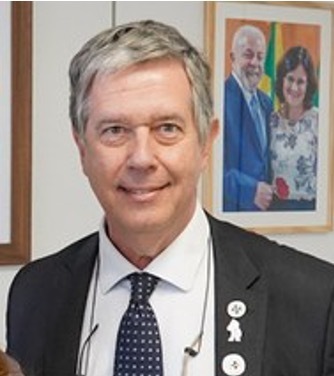

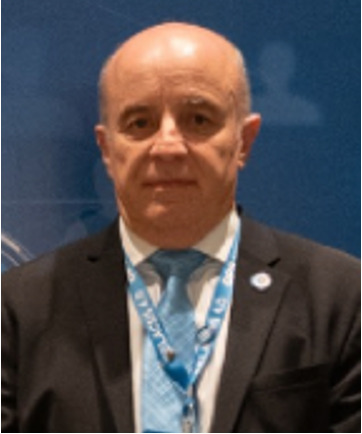

 The *Revista Latino-Americana de Enfermagem* (RLAE), a scientific dissemination vehicle of the *Escola de Enfermagem* of the *Universidade de São Paulo* in Ribeirão Preto and Collaborating Centre of the Pan American Health Organization and World Health Organization (PAHO/WHO) for Nursing Research Development, is aligned with global and national trends, publishing this special thematic edition “Digital health: contributions from Nursing”. 

 The selected articles address relevant and priority topics in the *Sistema Único de Saúde* (SUS), from a contemporary perspective and applied through emerging digital technologies and within the scope of digital health. Both the perspective of health professionals, with an emphasis on Nursing, and the user are considered, such as the article that deals with a serious game aimed at teenagers, with the theme of safe sex and contraception ^(^ 1 ^)^ . Along the same lines, focusing on SUS users, is the article that addresses telenursing applied to the self-care of people with heart failure during the COVID-19 pandemic ^(^ 2 ^)^ . Also noteworthy is the scoping review of teleconsultation in Nursing ^(^ 3 ^)^ and the Nursing workload classification model, using artificial intelligence techniques ^(^ 4 ^)^ . 

 This edition of RLAE coincides with a key moment in which the *Secretaria de Informação e Saúde Digital* of the *Ministério da Saúde* (SEIDIGI/MS), created in early 2023 by the Minister of Health Nísia Trindade, has been leading the initial stages of implementing the *SUS Digital* Program ^(^ 5 ^)^ . The main objective of *SUS Digital* is to promote progress in the digital transformation of the Brazilian health system, guaranteeing its principles and therefore expanding the population’s access to health actions and services, reducing inequalities, creating conditions for continuity of care, and, at the same time, designing the application of new digital technologies with a view to ensuring their critical use and ethical principles in their application. 

 Within the scope of the SUS Digital Program, it is worth highlighting the *MeuSUS* *Digital* Application, which has been progressively gaining new functionalities and whose main innovation was to make the long-awaited “access to your own health data” for every Brazilian citizen possible. At the same time, continuity of care will be guaranteed by the patient’s electronic medical record, which will gradually be available at any point in the service network, during the patient’s care context. 

 Both applications became possible thanks to the interoperability architecture model adopted by the *Ministério da Saúde* , through the National Health Data Network (RNDS in Portuguese), capable of interoperating health data on a large scale and originating from different information systems, breaking with its historical fragmentation, which has always resulted in the difficulty of producing analyses that depend on the integration of databases to generate strategic information. 

 In a complementary dimension, the Open Data Plan (PDA in Portuguese) 2024-2026 has just been approved by the Digital Governance Committee, coordinated by the Executive Secretariat of the *Ministério da Saúde* . For the first time, the initial proposal was submitted to public consultation, and in 48 days received 662 suggestions from civil society, which were incorporated and will contribute to active transparency in the dissemination of strategic information by the Ministry. Open data consists of public databases, actively made available on the internet for general public use. Data is provided in a non-proprietary file format. This is an action that increases public transparency. 

 Led by SEIDIGI/MS, with the participation of all Secretariats of the *Ministério da Saúde* , PDA 2024-26 received contributions and institutional support, at all stages, from the Comptroller General of the Union. The PDA is a legal commitment established by Decree nº 8,777/2016 ^(^ 6 ^)^ , which establishes the Federal Executive’s Open Data Policy. As data is available in an open format, anyone can access and use it without the need for proprietary software, promoting active transparency and the use of this information by the entire society. 

 Currently, the Ministry’s Open Data Portal ^(^ 7 ^)^ has 40 open databases. And, during the PDA-MS inventory, it was pointed out that there are 229 databases that could be opened. The goal is that, by the end of 2024, the ministerial office will have 86 open databases, doubling the present availability. It is important to highlight that all data that goes to the Open Data Portal is subjected to a de-identification and anonymization process, in compliance with the General Data Protection Law (LGPD in Portuguese) ^(^ 8 ^)^ . 

 These and other measures that represent advances in the structuring of an information and digital health policy became possible with the creation of SEIDIGI. The Secretariat’s work has been monitored and supported by the Pan American Health Organization (PAHO), with which the Brazilian *Ministério da Saúde* signed the first Cooperation Agreement for the Americas (TC 157) with a scope in digital health. PAHO has mentioned that the progress Brazil has been making could inspire other countries in the region to follow the same path, contributing to the strengthening of health systems, especially those that are public and universally accessible, such as the SUS. 
