# Supplementary material for: Digital health: contributions from Nursing
Source: Rev Lat Am Enfermagem. 2024 Nov 4;32:e4407. doi: 10.1590/1518-8345.0000.4407 (PMC11537260; doi:10.1590/1518-8345.0000.4407)
Supplement: Translation

# Translation: Salud digital: aportes desde la Enfermería

**DOI:** 10.1590/1518-8345.0000.4406

**Published:** 2024-10-16

**Authors:** Ana Estela Haddad, Swedenberger Barbosa, Paulo Eduardo Guedes Sellera, Marcelo D’Agostino

**Affiliations:** ^1^ Ministério da Saúde, Secretaria de Informação e Saúde Digital, Brasília, DF, Brasil; ^2^ Ministério da Saúde, Secretaria Executiva, Brasília, DF, Brasil; ^3^ Organização Pan-Americana da Saúde/Organização Mundial da Saúde, Departamento de Evidência e Inteligência para Ação em Saúde, Brasília, DF, Brasil

 La *Revista Latino-Americana de Enfermagem* (RLAE), vehículo de divulgación científica de la *Escola de Enfermagem* de la *Universidade de São Paulo* de Ribeirão Preto y Centro Colaborador de la Organización Panamericana de la Salud y la Organización Mundial de la Salud (OPS/OMS) para el Desarrollo de la Investigación en Enfermería, se alinea con la tendencia global y nacional, publicando esta edición especial temática “Salud digital: aportes desde la Enfermería”. 

 Los artículos seleccionados abordan temas relevantes y prioritarios en el *Sistema Único de Saúde* (SUS), desde una perspectiva contemporánea y aplicada por medio de tecnologías digitales emergentes y en el ámbito de la salud digital. Tanto la óptica de los profesionales de la salud, con énfasis en Enfermería, como la del usuario están en perspectiva, como por ejemplo, el artículo que trata sobre un *serious game* dirigido a adolescentes, con temática de sexo seguro y anticoncepción ^(^ 1 ^)^ . En la misma línea, centrado en el usuario del SUS, se encuentra el artículo que aborda la teleenfermería aplicada al autocuidado de personas con insuficiencia cardíaca durante la pandemia de COVID-19 ^(^ 2 ^)^ . Destaca también la revisión de alcance de la teleconsulta en Enfermería ^(^ 3 ^)^ y el modelo clasificador de carga de trabajo en Enfermería, utilizando técnicas de inteligencia artificial ^(^ 4 ^)^ . 

 Esta edición de la RLAE coincide con un momento clave en el que la Secretaría de Información y Salud Digital del *Ministério da Saúde* (SEIDIGI/MS), creada a principios de 2023 por la Ministra de Salud Nísia Trindade, viene liderando las etapas iniciales de implementación del Programa *SUS Digital* ^(^ 5 ^)^ . El principal objetivo del *SUS Digital* es promover el avance en la transformación digital del sistema de salud brasileño, garantizando sus principios y, por tanto, la ampliación del acceso de la población a las acciones y servicios de salud, la reducción de inequidades, la creación de las condiciones para la continuidad del cuidado y, al mismo tiempo, diseñando la aplicación de las nuevas tecnologías digitales desde la perspectiva de asegurar su uso crítico y los principios éticos en su aplicación. 

 En el ámbito del Programa *SUS Digital* , cabe destacar la Aplicación *MeuSUS Digital* , que progresivamente viene ganando nuevas funcionalidades y cuya principal innovación fue brindar el tan ansiado “acceso a sus propios datos de salud” para cada ciudadano y ciudadana brasileños. Al mismo tiempo, la continuidad del cuidado estará garantizada por medio de la historia clínica electrónica del paciente, que estará disponible gradualmente en cualquier punto de la red de servicios, durante el contexto de atención del paciente. 

 Ambas aplicaciones fueron posibles gracias al modelo de arquitectura de interoperabilidad adoptado por el *Ministério da Saúde* , por medio de la Red Nacional de Datos en Salud (RNDS), capaz de interoperar datos de salud a gran escala y provenientes de diferentes sistemas de información, rompiendo con su fragmentación histórica, que siempre ha resultado en la dificultad de producir análisis que dependan de la integración de bases de datos para generar información estratégica. 

 En una dimensión complementaria, el Plan de Datos Abiertos (PDA) 2024-2026 acaba de ser aprobado por el Comité de Gobernanza Digital, coordinado por la Secretaría Ejecutiva del *Ministério da Saúde* . Por primera vez, la propuesta inicial fue sometida a consulta pública, y en 48 días recibió 662 sugerencias, provenientes de la sociedad civil, las cuales fueron incorporadas y contribuirán a la transparencia activa en la difusión de información estratégica por parte del Ministerio. Los datos abiertos consisten en bases de datos públicas, disponibles activamente en Internet para uso del público en general. Los datos están disponibles en un formato de archivo no propietario. Esta es una acción que aumenta la transparencia pública. 

 Realizado por la SEIDIGI/MS, con la participación de todas las Secretarías del *Ministério da Saúde* , el PDA 2024-26 recibió aportes y apoyo institucional, en todas sus etapas, de la Contraloría General de la Unión. El PDA es un compromiso legal establecido por el Decreto nº 8.777/2016 ^(^ 6 ^)^ , que establece la Política de Datos Abiertos del Ejecutivo Federal. Al estar los datos disponibles en formato abierto, cualquier persona puede acceder a ellos y utilizarlos sin necesidad de disponer de un *software* propietario, promoviendo la transparencia activa y el uso de esta información por toda la sociedad. 

 Actualmente, el Portal de Datos Abiertos ^(^ 7 ^)^ del Ministerio cuenta con 40 bases de datos abiertas. Y, durante el inventario del PDA-MS, se señaló que existen 229 bases de datos que se pueden abrir. El objetivo es que, a finales de 2024, el despacho ministerial cuente con 86 bases de datos abiertas, duplicando la disponibilidad actual. Es importante resaltar que todos los datos que van al portal de Datos Abiertos pasan por un proceso de desidentificación y anonimización, de acuerdo con la Ley General de Protección de Datos (LGPD) ^(^ 8 ^)^ . 

 Estas y otras medidas que representan avances en la estructuración de una política de información y salud digital fueron posibles con la creación de la SEIDIGI. El trabajo de la Secretaría ha sido seguido y apoyado por la Organización Panamericana de la Salud (OPS), con la que el *Ministério da Saúde* de Brasil firmó el primer Acuerdo de Cooperación de las Américas (TC 157) con alcance en salud digital. La OPS ha mencionado que los avances que viene logrando Brasil podrían inspirar a otros países de la región a seguir el mismo camino, contribuyendo al fortalecimiento de los sistemas de salud, especialmente aquellos que son públicos y tienen acceso universal, como el SUS. 

# Translation: Saúde digital: contribuições da Enfermagem

**DOI:** 10.1590/1518-8345.0000.4408

**Published:** 2024-10-16

**Authors:** Ana Estela Haddad, Swedenberger Barbosa, Paulo Eduardo Guedes Sellera, Marcelo D’Agostino

**Affiliations:** ^1^ Ministério da Saúde, Secretaria de Informação e Saúde Digital, Brasília, DF, Brasil; ^2^ Ministério da Saúde, Secretaria Executiva, Brasília, DF, Brasil; ^3^ Organização Pan-Americana da Saúde/Organização Mundial da Saúde, Departamento de Evidência e Inteligência para Ação em Saúde, Brasília, DF, Brasil

A Revista Latino-Americana de Enfermagem (RLAE), veículo de divulgação científica da Escola de Enfermagem da Universidade de São Paulo de Ribeirão Preto e Centro Colaborador da Organização Panamericana da Saúde e Organização Mundial de Saúde (OPAS/OMS) para o Desenvolvimento da Pesquisa em Enfermagem, coloca-se alinhada com tendência global e nacional, publicando esta edição especial temática “Saúde digital: contribuições da Enfermagem”.

 Os artigos selecionados abordam temas relevantes e prioritários no Sistema Único de Saúde (SUS), sob a perspectiva contemporânea e aplicada por meio de tecnologias digitais emergentes e sob o escopo da saúde digital. Estão em perspectiva tanto a ótica dos profissionais de saúde, com ênfase na Enfermagem, como também a do usuário, como por exemplo, o artigo que trata de um *serious game* voltado para adolescentes, com o tema do sexo seguro e contracepção ^(^ 1 ^)^ . Na mesma linha, do foco no usuário do SUS, está o artigo que aborda a telenfermagem aplicada ao autocuidado de pessoas com insuficiência cardíaca durante a pandemia da COVID-19 ^(^ 2 ^)^ . Destaca-se também a revisão de escopo sobre a teleconsulta na Enfermagem ^(^ 3 ^)^ e o modelo classificador da carga de trabalho da Enfermagem, utilizando técnicas de inteligência artificial ^(^ 4 ^)^ . 

 Esta edição da RLAE coincide com momento-chave em que a Secretaria de Informação e Saúde Digital do Ministério da Saúde (SEIDIGI/MS), criada no início de 2023, pela Ministra da Saúde Nísia Trindade, vem conduzindo as etapas iniciais de implementação do Programa SUS Digital ^(^ 5 ^)^ . O SUS Digital tem como principal objetivo promover o avanço na transformação digital do sistema brasileiro de saúde, garantindo os seus princípios e portanto a ampliação do acesso da população às ações e serviços de saúde, a redução de iniquidades, a criação das condições para a continuidade do cuidado, e, ao mesmo tempo, desenhando a aplicação das novas tecnologias digitais na perspectiva de garantir o seu uso crítico e os princípios éticos na sua aplicação. 

No âmbito do Programa SUS Digital vale destacar o Aplicativo MeuSUS Digital, que vem ganhando progressivamente novas funcionalidades e cuja principal inovação foi efetivar o tão sonhado “acesso aos seus próprios dados de saúde” por cada cidadão e cidadã brasileiros. Ao mesmo tempo, a continuidade do cuidado será garantida pelo prontuário eletrônico do paciente que estará gradualmente disponível em qualquer ponto da rede de serviços, durante o contexto de atendimento do paciente.

Ambos os aplicativos se tornaram possíveis graças ao modelo de arquitetura de interoperabilidade adotado pelo Ministério da Saúde, por meio da Rede Nacional de Dados em Saúde (RNDS), capaz de interoperar dados de saúde em grande escala e oriundos de diversos sistemas de informação, rompendo com a sua histórica fragmentação, que sempre resultou na dificuldade de produzir análises que dependem da integração de bases de dados para gerar informações estratégicas.

Em dimensão complementar, acaba de ser aprovado pelo Comitê de Governança Digital, coordenado pela Secretaria Executiva do Ministério da Saúde, o Plano de Dados Abertos (PDA) 2024-2026. Pela primeira vez, a proposta inicial foi colocada em consulta pública e em 48 dias recebeu 662 sugestões, advindas da sociedade civil, que foram incorporadas, e contribuirão para a transparência ativa na disseminação de informações estratégicas pelo Ministério. Dados abertos consistem em bases de dados públicos, disponibilizadas de modo ativo na internet para uso público em geral. Os dados são disponibilizados em formato de arquivo que não é proprietário. Esta é uma ação que aumenta a transparência pública.

 Conduzido pela SEIDIGI/MS, com a participação de todas as Secretarias do Ministério da Saúde, o PDA 2024-26 recebeu contribuições e o apoio institucional, em todas as suas etapas, da Controladoria Geral da União. O PDA é compromisso legal instituído pelo Decreto nº 8.777/2016 ^(^ 6 ^)^ , que estabelece a Política de Dados Abertos do Executivo Federal. Com os dados disponibilizados em formato aberto, qualquer pessoa pode acessar e utilizar os dados sem a necessidade de possuir um *software* proprietário, promovendo a transparência ativa e a utilização dessas informações por toda a sociedade. 

 Atualmente, o Portal de Dados Abertos ^(^ 7 ^)^ do Ministério conta com 40 bases de dados abertas. E, durante o inventário do PDA-MS, foi apontado que há 229 bases de dados possíveis de serem abertas. A meta é que, até o final de 2024, a pasta tenha 86 bases de dados abertas, dobrando a disponibilidade atual. É importante ressaltar que todos os dados que vão para o Portal de Dados Abertos passam por um processo de desidentificação e anonimização, em conformidade com a Lei Geral de Proteção de Dados (LGPD) ^(^ 8 ^)^ . 

Essas e outras medidas que representam avanços na estruturação de uma política de informação e saúde digital tornaram-se possíveis a partir da criação da SEIDIGI. A atuação da Secretaria tem sido seguida e apoiada pela Organização Panamericana da Saúde (OPAS), com a qual o Ministério da Saúde do Brasil firmou o primeiro Termo de Cooperação das Américas (TC 157) com escopo na saúde digital. A OPAS tem mencionado que os avanços que o Brasil vem fazendo poderão inspirar outros países da região a seguir o mesmo caminho, contribuindo para o fortalecimento dos sistemas de saúde, em especial aqueles que são públicos e de acesso universal como é o caso do SUS.
